# COVID-19 – A Covert Catalyst for Pedagogical Stocktake and Transformation: Perspectives of a Global Hub

**DOI:** 10.15694/mep.2020.000212.1

**Published:** 2020-09-29

**Authors:** Mei Li Khong, Enoch Chan, Julian Alexander Tanner, Pamela Pui Wah Lee, Gordon Wong

**Affiliations:** 1 The University of Hong Kong

**Keywords:** e-learning, COVID-19, modernization, blended learning, undergraduate medical education, pandemic preparedness, disruptions, webside teaching, wellbeing, telemedicine

## Abstract

This article was migrated. The article was marked as recommended.

Education in Hong Kong was shifted online on an abrupt and massive scale as the city faced unprecedented disruptions, first from social unrest in 2019 and then again with the COVID-19 pandemic. Concurrent modernization initiatives since early 2019 in The University of Hong Kong’s Medical Faculty (HKUMed), conferred a fortuitous head start for this rapid change. Pre-clinical and clinical teaching were restructured for online delivery through e-learning solutions for didactic teaching, and new innovative approaches were developed to convert bedside to“webside” teaching. E-learning in the current circumstances provided necessary social distancing while being pedagogically sound. Students were also able to develop key communication and collaboration skills via online platforms, developing digital skills critical to their future profession. However, unforeseen issues including socioeconomic inequality, privacy concerns, and social isolation became apparent and should be addressed as medical education progresses further down the digital path. The goal must entail a sustainable and scholarly approach towards optimizing medical education in an increasingly online environment. This will help safeguard the medical curriculum against disruption and empower future medical professionals for tomorrow’s practice. Here, we share experiences and perspectives from educators in a global city characterised by land scarcity and income inequality.

## Introduction

The recent pandemic has driven people worldwide into periods of rapid adjustments which provide unprecedented opportunities to reflect on a personal, communal, corporate, and even on a national or global level. Those further along on the continuum of COVID-19 pandemic may have begun contemplating the transition back to normalcy. The confluence of seemingly serendipitous factors, including an unrelated push towards e-learning, an unfortunate head start to the class disruption and a relatively expeditious flattening of the curve, has placed us at The University of Hong Kong Medical Faculty (HKUMed) in a privileged position to be able to share some longer-term perspectives as well as lessons learnt. While some thoughts are obviously of more local and contemporaneous relevance, the bulk we would consider transcends geographical and temporal borders.

## Hong Kong’s Geo-socio-political Sphere and Medical Education

Given its position as an international travel and economic hub, Hong Kong is not estranged to havoc arising from emerging infections. During the SARS epidemic in 2002 ( Peng *et al.*, 2003), Hong Kong was hit with the second highest number of cases and deaths worldwide ( World Health Organization, 2003). Later we also played host to H1N1 swine influenza, Middle East Respiratory Syndrome (MERS), avian influenza amongst others. While land is sparse in most of mainland China, Hong Kong is geographically different. The city’s land scarcity and income inequality have pushed many people into high density living arrangements. Public transport systems, though very efficient, are often terribly crowded as are other places of public gathering. Together these characteristics create an inviting milieu for infectious diseases to reach epidemic proportions. Cognizant of this potential, the local government and health authorities has a low threshold in instituting measures aimed at disrupting the transmission of infectious diseases.

Hong Kong’s proximity to mainland China both geographically and economically means daily bilateral movement of people across the border is usually substantial. When the news of a novel coronavirus outbreak in Wuhan reached Hong Kong, our local government was poised to take necessary action. Our Hospital Authority, the body responsible for governing the public hospitals swiftly raised emergency levels in late January 2020, implementing restricted access to clinical areas for non-essential people, including health care students. With the Chinese New Year holiday period approaching at that time, an early break from school was instituted and face-to-face classes were suspended since. Coincidently city-wide social unrests throughout 2019, which at one period escalated to violent and destructive protests, including the occupation of two university campuses, had led to class suspension in November 2019 due to safety concerns and public transport disruption. Therefore, our students and teachers had been dealing with class disruption for a far longer period than those outside Hong Kong and mainland China.

## Our E-learning Journey

Quite independently of COVID-19, HKUMed was facing expectant large increases in class sizes across multiple programs, due to the need for more medical or healthcare graduates to cope with the aging population in the city. However, there is very limited potential for physical space expansion. Thus, we were compelled to explore pedagogically sound and upscalable strategies that are less dependent on physical space. At the start of 2019, we moved to progressively transform a large portion of the MBBS curriculum into a blended learning format, while concurrently modernizing its content to prepare our graduates for future healthcare practices. This initiative kicked off with the construction of a small but well-equipped recording studio with technical support that commenced operation from April 2019. Progressively, we increased production support to include pre-shooting guidance, personalized on-site technical assistance, and post-production video editing which enabled us to upscale the production of high-quality e-learning material. From September 2019 onwards, we implemented blended learning in our curriculum by incorporating lecture videos supplemented with formative assessments containing built-in feedback into approximately 50% of the pre-clinical curriculum. Online forums within the learning management system also enabled student-student and student-teacher interactions. We also understood the need to ensure that e-learning was visually and aurally accessible to all students with different needs and constraints. Therefore, we prioritized specific approaches to suit these needs such as recording all face-to-face lectures and adding closed captioning to instructional videos.

After the class suspensions in late 2019, we became confident that e-learning could sustain student learning even during class disruptions. Asynchronous e-learning activities provided students the flexibility to engage with the material at their convenience and in a safe space. Teachers benefitted from learning analytics that charted students’ learning engagement. Staff engagement with e-learning was enabled by clear guidelines and demonstration videos, providing them with a choice of different modalities of production coupled with back-end support.

However, moving from physical classes to e-learning raised issues related to access and equity. We needed to ensure teachers and students had the basic technology and support to conduct e-learning. To further strengthen these initiatives, we equipped teachers and students to use online tools effectively. HKUMed’s learning designers provided teachers with pedagogical support on delivering blended learning through harnessing e-learning videos, current educational technology, and innovative teaching approaches. Students were also provided with remote mentorship and coaching to support their learning through online platforms.

## Arrival of COVID-19

As the COVID-19 pandemic unfolded, it became apparent that educational activities had to be shifted online on a scale and suddenness none had envisaged. With the access to clinical areas abruptly stopped, our normally well-integrated medical curriculum had to be restructured rapidly. The didactic components had to be distilled into video lectures, while clinical teaching and practical classes were desynchronized from corresponding didactic teaching. Clinical teachers that were previously unenthusiastic with e-learning became more inclined to change and explore innovative ways to sustain clinical teaching. Novices to e-learning benefitted from the clear written guidelines and demonstration videos developed from the pre-clinical curriculum experience. Several recording options with different prerequisites for previous experience, familiarity with recording software and recording location were offered to them. Using interactive video conference technology ( Tsang *et al.*, 2020), clinical teachers also began online “bedside” tutorials (i.e. “webside”) with stable patients at designated areas with increased privacy and Wi-Fi capacities. Although this was no replacement for learning physical examinations at the bedside, it provided the necessary avenue to facilitate clinical discussions and educate students on professional bedside / “webside” etiquette. Some clinical teachers adopted a three-pronged approach to replace face-to-face clinical demonstrations by introducing video-based and written guidelines followed by online small group tutorials. Shih and colleagues ( Shih *et al.*, 2020) reported that this teaching method encouraged students to take a more proactive role in self-directed learning through pre-class materials as well as online small group tutorials for the critical reinforcement of self-directed learning.

## Looking Backwards and Around before Moving Forwards

From our experience with transition to e-learning during disruptive times, we recognized the importance of engraving an e-learning culture within the Medical Faculty. Any new infrastructure or expertise requires time to develop and be accepted within an organization. From a recent Faculty survey conducted, those who felt less equipped for e-learning had a more negative experience with this transition. As a result of our incidental preparedness in e-learning, HKUMed was enabled to make the rapid shift to online medical education without much difficulties. With the COVID-19 outbreak coming under control and the easing of social distancing measures, we have to plan towards transitioning back to the new normal. We must ponder whether pedagogically we should, or even could return to status quo or we must evolve in a different direction. In other words, will we merely transition back or transform forwards? Though the jury is still out, it would be prudent to examine what we and others have learnt in order to move forward.

1. Even when hands are forced, we still need to deal with human factors

While necessity may be the mother of invention, necessity also compels one to confront their prejudices and reservations towards change. Understandably, teachers are heterogenous in terms of their background, their digital literacy and comfort with being online. We cannot assume the same level of pre-existing e-learning competencies across all teachers and students. Reluctance to be recorded may stem from the fear of being held accountable for every word spoken in a recorded lecture, with one’s words being repeatedly analysed and even have the potential to be widely disseminated for scrutiny. However, faculty members were left with few options other than deferring or cancellation of teaching. Given the unpredictable duration of disruption and whether classes can be made up in the future, most staff did engage in e-learning and as they did, most of their concerns seemed to have dissipated.

2. Social equity and wellbeing issues with large scale e-learning

In terms of students’ wellbeing, learning at home results in isolation. The freedom to withdraw from social interaction during synchronous learning activities by turning off one’s audio and video compounds this issue. It is important to keep in mind that the university is a social environment, in which students within a program of study share a common pursuit. Their academic success is dependent on social interactions (such as peer-to-peer support, feedback and positive competitions) that promote their learning process as well as their sociopsychological wellbeing.

Socioeconomic inequality and privacy issues are also exposed as the quality of real-time webcasts is dependent on computing power and internet speed. Virtual backgrounds were unavailable to some with less powerful computers, forcing students either to display their home environment for all to see or to switch off the video display, thus creating a false sense of non-participation that may eventuate into real non-participation. Audio participation is also easily perturbed in Hong Kong, since many students reside in smaller apartments and are hence forced to remain in close proximity to other household members, leaving them prone to disturbance.

To address these issues, we must ensure students have the essential means to learn remotely. Some responses from other institutions include offering stipends for internet access and laptop rentals / purchases; loaning computing equipment; and providing internet hotspots for under-resourced students ( Heitz *et al.*, 2020). It is also necessary to expand mental health services on the medical campus for students ( Hall *et al.*, 2020). Due to social distancing measures, HKUMed scaled up outreach with individual/group counseling services and online mindfulness classes as well as key mental health resources to enable faculty and staff to support students. Given the distress caused by COVID-19, we see the ongoing need for it to grow for students, faculty, and staff alike.

Moving forward we should be cognizant of the fact that technological firepower should not determine one’s access to quality teaching. While up-market e-learning tools may be available, it should not exceed the hardware requirement affordable by the average income student and be accessible to all students across different socioeconomic backgrounds. When designing learning spaces, faculties should consider the installation of decent hardware to ensure optimal access to online learning.

3. What to keep and what to discard

While social distancing measures may soon relax, the speed and degree at which it should take place and the aspects of social distancing that are to remain are still uncertain. The million-dollar question is to know what elements of the pedagogical changes to keep and what to discard. *What if social distancing is here to stay?* The same Faculty survey revealed strong opinions regarding how e-learning cannot replace face-to-face teaching. Teachers missed the interactions and the immediate feedback when delivering a face-to-face lecture. However, apart from the tactile component, what are the other key aspects of face-to-face teaching that teachers feel irreplaceable? Is the chasm between face-to-face delivery and online delivery irreconcilable? What is best taught online and what is best taught face-to-face?

4. Changes in societal expectations

Doctors, patients, medical students, non-clinical and clinical teachers previously may have been less open to e-learning as face-to-face interaction was regarded as the norm and irreplaceable. However, we are now at a unique juncture in the history of medical practice where genomics, biosensors, electronic patient records, and artificial intelligence have all become superimposed on a digital infrastructure. Eric Topol opines that this remarkable set of technology promises healthcare delivery in a far more rational, efficient and tailored manner ( Topol, 2019a; Topol, 2019b). However, are our future medical professionals and teachers sufficiently digitally literate for this new era of e-medicine? What are the societal attitudes towards the increasingly digital forms of medical education and medical care?

Since the rapid shift to e-learning, most of our HKUMed students view e-learning as superior in providing flexibility and convenience for didactic teaching. The benefits of asynchronous learning (i.e. the ability to pause videos to take notes, annotate videos in real-time, re-watch videos, adjust video speed, and learn medical / scientific terms from closed captioning) are far-reaching and enable our students to learn more effectively than face-to-face delivery. Asynchronous learning (and perhaps assessment) may pave the way towards achieving competency rather than time-based paradigm of medical training.

A main drawback is the technical issues involved with e-learning. As it is likely that videoconferencing will remain a popular choice for clinical teaching even after COVID-19 pandemic, it is even more important for aspiring health care professionals to develop active engagement skills such as active listening, exchanging ideas, reaching consensus, and problem solving in both physical and online environments. In effect, engaging students for deep learning in a collaborative virtual classroom setting will prepare them for a collaborative virtual workplace in the future, connecting professionals to solve patient / service / administrative issues. The gap is how to equip teachers with sufficient pedagogical skills in the virtual learning environment.

Moving forward, we may need to embark upon changing societal expectations regarding the future virtual outlook of doctor-patient, doctor-doctor, teacher-student and student-student relations. We see the exigency of boosting medical students’ digital literacy to prepare them for the workforce. The move to an online learning environment is a key step in developing skills and changing attitudes that are required to become both digitally competent and confident. It is also important to create curriculum space to accommodate teaching of new medical knowledge essential in tomorrow’s clinical practice (e.g. Precision Medicine, PanorOmics, big data, artificial intelligence).

An emerging application of technology is telemedicine which involves the provision of health care remotely using telecommunication and information technology while delivering the same standard of care compared to face-to-face consultations. Within primary care, there is increasing evidence showing the benefits of telemedicine in providing ease of access for patients while reducing non-attendance rates ( The Health Foundation, 2014; Farr *et al.*, 2018; Marshall, Shah and Stokes-Lampard, 2018). Video consultations are well-received especially for patients who require long-term care (NHS England, 2016). Another application of telemedicine is secure video camera monitoring of patients in care homes by healthcare providers in order to give medical advice to care home staff and residents ( Airdale NHS Foundation Trust, 2020). Computer vision and machine learning algorithms also enable medical professionals to plan care proactively through early detection of adverse events such as falls ( de Miguel *et al.*, 2017). These technological advances confirm the need to develop more “webside” medical education ( Tsang *et al.*, 2020).

## Conclusions

Recent events have forcibly propelled some of us well into the 4 ^th^ industrial revolution, accelerated our e-learning development and highlighted the need for suitable infrastructure and expertise. Locally, we must be ever ready for disruption to classes especially with the history of social unrests and the susceptibility of Hong Kong towards importation of emerging infectious diseases. While e-learning may not be the panacea, we need to look beyond the immediate crisis. We must not mistake disruption-driven innovations for pedagogical superiority. It is pivotal to establish a scholarly, sustainable and forward-thinking approach to understand how best to facilitate student learning in an online environment. The COVID-19 crisis provided an opportunity for medical education institutions to experiment and innovate. Piloting approaches and building on effective practices will enable positive and enduring changes that not only confer immunity to further disruptions, but also prepare doctors to be competent practitioners in both the physical and online environments in this 21 ^st^ century.

## Take Home Messages

• A global hub like Hong Kong is particularly prone to epidemics which may cause disruptions to educational systems.
• Disruptions pushed existing e-learning solutions beyond their boundaries in replacing a vast majority of face-to-face teaching.
• Disruption-driven innovations should not be mistaken as pedagogical superiority and therefore, critical reflections on various issues are warranted.
• Medical curriculum should adapt according to the future outlook of modern medicine and boost medical students’ digital literacy.
• E-learning provides resilience to the medical curriculum and prepares future graduates for a 21 ^st^ century career.

## Notes On Contributors

**Mei Li Khong**, PhD, is an E-learning Instructor, LKS Faculty of Medicine, The University of Hong Kong, Hong Kong. ORCID: https://orcid.org/0000-0003-0668-2949.

**Enoch Chan**, PhD, is an E-learning Instructor, LKS Faculty of Medicine, The University of Hong Kong, Hong Kong. ORCID: https://orcid.org/0000-0003-1213-3428.

**Julian Alexander Tanner**, PhD, FRSC, is a Professor and Associate Director (Teaching and Learning), School of Biomedical Sciences, LKS Faculty of Medicine, The University of Hong Kong; and Assistant Dean (Biomedical Sciences Curriculum), LKS Faculty of Medicine, The University of Hong Kong, Hong Kong. ORCID: https://orcid.org/0000-0002-5459-1526.

**Pamela Pui Wah Lee**, MBBS, MD, FHKCPaed, FHKAM (Paediatrics), is a Clinical Associate Professor, Department of Paediatrics and Adolescent Medicine, LKS Faculty of Medicine, The University of Hong Kong; Assistant Dean (Clinical Curriculum), LKS Faculty of Medicine, The University of Hong Kong; Programme Director (Pedagogy and Training), Bau Institute of Medical and Health Sciences Education, LKS Faculty of Medicine, The University of Hong Kong, Hong Kong. ORCID: https://orcid.org/0000-0002-5934-7338.

**Gordon Tin Chun Wong**, MBBS, BSc (Med), MD, FANZCA, FHKCA, FHKAM (Anaesthesiology), is a Clinical Associate Professor, Department of Anaesthesiology, LKS Faculty of Medicine, The University of Hong Kong; Honorary Associate Professor, Department of Pharmacology and Pharmacy, LKS Faculty of Medicine, The University of Hong Kong; Assistant Dean (Professional Development), LKS Faculty of Medicine, The University of Hong Kong; Programme Director (Innovation and Collaboration), Bau Institute of Medical and Health Sciences Education, LKS Faculty of Medicine, The University of Hong Kong, Hong Kong. ORCID: https://orcid.org/0000-0002-8127-4593.

## References

[ref1] Airdale NHS Foundation Trust (2020) Telemedicine (Digital Care Hub). Available at: http://www.airedale-trust.nhs.uk/services/telemedicine/( Accessed: 4 May 2020).

[ref2] de Miguel K. Brunete A. Hernando M. and Gambao E. (2017) Home Camera-Based Fall Detection System for the Elderly. Sensors (Basel). 17(12). 10.3390/s17122864 PMC5751723 29232846

[ref3] Farr M. Banks J. Edwards H. B. Northstone K. (2018) Implementing online consultations in primary care: a mixed-method evaluation extending normalisation process theory through service co-production. BMJ Open. 8(3), p. e019966. 10.1136/bmjopen-2017-019966 PMC5875620 29555817

[ref4] Hall A. K. Nousiainen M. T. Campisi P. Dagnone J. D. (2020) Training disrupted: Practical tips for supporting competency-based medical education during the COVID-19 pandemic. Med Teach.pp.1–6. 10.1080/0142159X.2020.1766669 32450049

[ref5] Heitz C. Laboissiere M. Sanghvi S. and Sarakatsannis J. (2020) Getting the next phase of remote learning right in higher education. Available at: https://www.mckinsey.com/industries/public-sector/our-insights/getting-the-next-phase-of-remote-learning-right-in-higher-education( Accessed: 28 April 2020).

[ref6] Marshall M. Shah R. and Stokes-Lampard H. (2018) Online consulting in general practice: making the move from disruptive innovation to mainstream service. BMJ. 360, p.k1195. 10.1136/bmj.k1195 29581174

[ref7] NHS England (2016) General Practice Forward View. Available at: https://www.england.nhs.uk/gp/gpfv/( Accessed: 4 May 2020).

[ref8] Peng G. W. He J. F. Lin J. Y. Zhou D. H. (2003) Epidemiological study on severe acute respiratory syndrome in Guangdong province. Zhonghua Liu Xing Bing Xue Za Zhi. 24(5), pp.350–2, https://www.ncbi.nlm.nih.gov/pubmed/12820925 12820925

[ref9] Shih K. C. Chan J. C. Chen J. Y. and Lai J. S. (2020) Ophthalmic clinical skills teaching in the time of COVID-19: A crisis and opportunity. Med Educ. 54(7), pp.663–664. 10.1111/medu.14189 32324929 PMC7264745

[ref10] The Health Foundation (2014) Shine: Improving the value of local healthcare services. Available at: https://www.health.org.uk/sites/default/files/ShineImprovingTheValueOfLocalHealthcareServices.pdf( Accessed: 6 May 2020).

[ref11] Topol E. J. (2019a) High-performance medicine: the convergence of human and artificial intelligence. Nat Med. 25(1), pp.44–56. 10.1038/s41591-018-0300-7 30617339

[ref12] Topol E. J. (2019b) Preparing the healthcare workforce to deliver the digital future. Available at: https://topol.hee.nhs.uk/wp-content/uploads/HEE-Topol-Review-2019.pdf( Accessed: 6 May 2020).

[ref13] Tsang A. C. O. Lee P. P. Chen J. Y. and Leung G. K. K. (2020) From bedside to webside: A neurological clinical teaching experience. Med Educ. 54(7), p.660. 10.1111/medu.14175 32285492 PMC7262309

[ref14] World Health Organization (2003) SARS epidemiology to date. Available at: https://www.who.int/csr/sars/epi2003_04_11/en/( Accessed: 6 May 2020).

