# Supplementary material for: COVID-19 – A Covert Catalyst for Pedagogical Stocktake and Transformation: Perspectives of a Global Hub
Source: MedEdPublish (2016). 2020 Sep 29;9:212. [Version 1] doi: 10.15694/mep.2020.000212.1 (PMC10699403; doi:10.15694/mep.2020.000212.1)
Supplement: Peer review history

# Reviewer report: Reviewer response for version 1

**DOI:** 10.21956/mep.20178.r31200

**Published:** 2020-12-18

**Authors:** Ken Masters

**Affiliations:** 1 Sultan Qaboos University

This review has been migrated. The reviewer awarded 4 stars out of 5 A really interesting, balanced and in-depth look at the impact of the COVID-19 pandemic on medical education and learning approaches in Hong Kong. The authors' frank account of the impact of the pre-COVID-19 disruptions, and the reflection on their activities is impressive. While the specifics prevent some of the lessons from being generalisable, other medical schools would be able to benefit from the lessons learned in the paper, particularly in the preparation and support processes.Two minor language points: • “While land is sparse in most of mainland China” Did the authors intend to write this, or did they mean “sparsely populated”?• “Audio participation is also easily perturbed” Perhaps “disturbed” is a better word here than “perturbed”Overall, a good and interesting read.

Reviewer Expertise:

NA

No decision status is available

# Reviewer report: Reviewer response for version 1

**DOI:** 10.21956/mep.20178.r31201

**Published:** 2020-10-07

**Authors:** Hui Meng Er

**Affiliations:** 1 International Medical University

This review has been migrated. The reviewer awarded 4 stars out of 5 The paper offers multiple perspectives in the efforts to safeguard medical education against disruption based on first-hand experience. The innovation also aims to empower future medical professionals for tomorrow’s practice.The practical considerations and approaches for transformation of face to face teaching and learning activities to e-Learning are well summarised. The emphasis on a scholarly approach, taking into consideration of institutional readiness (both faculty and students) is indeed important in order to ensure sustainability. It is a good reference for health professions educators moving forward beyond the Covid-19 pandemic.

Reviewer Expertise:

NA

No decision status is available
